# Retraction: Selective Molecular Alterations in the Autophagy Pathway in Patients with Lewy Body Disease and in Models of α-Synucleinopathy

**DOI:** 10.1371/journal.pone.0313935

**Published:** 2024-11-13

**Authors:** The PLOS ONE Editors

Following the publication of this article [1], concerns were raised regarding results presented in Figs 1, 2, 3, 5, 6, 8, S1, S2, and S3. Specifically,

- There appear to be similarities between lanes within numerous panels presented in:
  ○ Figs 1A and 1B
  ○ Figs 5A and 5B
  ○ Fig 8O
  ○ Figs S1A and S1B
  ○ Figs S2A and S2B
  ○ Fig S3D
- The Fig 6H and Fig 8H panels appear to partially overlap.

Regarding the similarities between and within the western blot panels, the corresponding author stated that the quality of the western blot panels presented in the article is not optimal and that close examination of these panels shows subtle differences between the lanes and bands that appear similar. The corresponding author provided some underlying image data but the editorial assessment was limited by the low quality of the images. PLOS remains concerned that the areas in the panels appear more similar than would be expected from independent results, and in absence of uncropped high resolution image data, these concerns cannot be resolved.

Regarding the panel overlap between Figs 6H and 8H, the corresponding author stated that an inadvertent error was made during figure preparation and they provided a replacement figure.

In light of the issues with the western blot panels that raise concerns about the reliability and integrity of the published results, the *PLOS ONE* Editors retract this article.

EM did not agree with the retraction. LC and PD responded but expressed neither agreement nor disagreement with the editorial decision. BS, CP, AP, ER, LH, AA, and DG either did not respond directly or could not be reached.
